# Tenax‐Based Electrospun Fibrous Membranes for Efficient VOC Sampling

**DOI:** 10.1002/marc.202400969

**Published:** 2025-03-03

**Authors:** R. Vilensky, O. Marom, D. M. Rein, E. Zussman

**Affiliations:** ^1^ Department of Mechanical Engineering Technion – Israel Institute of Technology Haifa 320004 Israel

**Keywords:** electrospinning, gas adsorption, membrane, polymer, VOC

## Abstract

The influence of solvent mixtures on electrospinning polymer solutions plays a crucial role in both the electrospinning process and the characteristics of the resulting fibers. Solvents with varying properties can be used to optimize thermodynamic stability, viscosity, evaporation rate, and dielectric constant of a solution simultaneously. This study investigates the fabrication of highly porous poly‐2,6‐diphenyl‐p‐phenylene oxide (PPPO) fibers via electrospinning from binary and ternary solvent mixtures. The resulting fibers exhibit significant porosity both at the surface and in the core, as evidenced by their high surface area and textured morphology. These features render the fibers highly effective for sampling volatile organic compounds (VOCs). Adsorption performance is evaluated under passive and dynamic conditions using Musk Xylene as a simulant. Compared to granular PPPO, the fibrous membranes achieved nearly a fourfold increase in passive adsorption and an sevenfold increase in dynamic adsorption. These findings highlight the potential of PPPO electrospun fibers as superior materials for VOC sampling applications.

## Graphical abstract

The study investigates the effects of solvent mixtures on the electrospinning of poly‐2,6‐diphenyl‐p‐phenylene oxide (PPPO) solutions and the characteristics of the resulting fibers. By optimizing binary and ternary solvent systems, highly porous fibers with controlled intrinsic and surface morphology can be produced. In dynamic conditions, the resulting fibers and mats enhance volatile organic compounds (VOCs) adsorption sevenfold over granular PPPO, demonstrating their effectiveness for high‐flow VOC sampling.

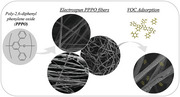

## 1 Introduction

The sampling of volatile organic compounds (VOCs) is of high importance in a variety of disciplines such as environmental sciences,^[^ ^1^, ^2^, ^3^ ^]^ clinical,^[^ ^4^, ^5^ ^]^ food and flavor^[^ ^6^ ^]^ and forensic sciences.^[^ ^7^, ^8^, ^9^, ^10^ ^]^ The conventional technique for air sampling and pre‐concentration of VOCs relies on the adsorption of the solute onto or into a solid adsorbent material.^[^ ^11^, ^12^, ^13^, ^14^ ^]^ The adsorbed solutes are then desorbed by liquid extraction or thermal desorption and subsequently transferred for analysis using various analytical equipment, such as ion mobility spectrometry (IMS),^[^ ^7^ ^]^ gas chromatography‐mass spectrometry (GC‐MS),^[^ ^15^ ^]^ or thermal desorption gas chromatography‐mass spectrometry (TD‐GC‐MS).^[^ ^3^, ^16^ ^]^

The adsorption of VOCs is governed by several key factors related to adsorbent material, the VOCs physical and chemical properties, and the ambient conditions. The critical properties of adsorbent materials include specific surface area, pore volume, pore size distribution, and surface chemistry. In the absence of specific interactions with VOC molecules, adsorption primarily depends on non‐specific interactions, such as van der Waals forces. In this case, the adsorption performance is mainly determined by the adsorbent pore structure and size distribution, where different pore types: micropores (<2 nm), mesopores (2–50 nm), and macropores (>50 nm) – each contributing uniquely to the adsorption process. The micropores are known to dominate adsorption capacity, while mesopores and macropores are crucial for enhancing the adsorption kinetics.^[^ ^17^, ^18^, ^19^, ^20^ ^]^ The primary adsorption mechanism in micropores is pore filling, where VOC molecules condense within the confined spaces of the micropores, whereas mesopores and macropores facilitate surface adsorption, either as a monolayer or in multiple layers. Additionally, interconnected pores improve gas/fluid transport and the permeability of adsorbent. Accordingly, a higher content of micropores increases the adsorption and retention capabilities, while meso‐ and macro‐pores facilitate the mass transfer of VOCs within the adsorbent. Furthermore, while the microporous structure is beneficial for enhancing adsorption capacity, it adversely affects desorption efficiency due to high mass‐transfer resistance. Hence, to optimize the adsorbent performance for various specific applications, the fabrication of hierarchically porous structures, including micropores, mesopores, and macropores, is often considered.^[^ ^18^, ^21^ ^]^

Porous organic polymers are one of the commercially available groups of adsorbent materials widely used for VOC sampling purposes. Tenax (TenaxTA) is a well‐known trademark of a poly‐2,6‐diphenyl‐p‐phenylene oxide (PPPO)^[^ ^22^, ^23^ ^]^ and is considered to be the leading porous polymer for air analysis.^[^ ^4^, ^6^, ^12^, ^13^, ^14^, ^15^, ^24^, ^25^ ^]^ It is widely utilized for VOCs sampling due to its high thermal stability, low chemical reactivity, and strong adsorption capacity for a broad range of VOCs, specifically nonpolar and slightly polar compounds within the analytical window.^[^ ^11^, ^26^, ^27^, ^28^ ^]^ Its key advantages include selective adsorption of low and medium‐volatility compounds while minimizing water adsorption, making it particularly suitable for humid environments.^[^ ^12^, ^27^ ^]^ These properties are critical for accurately capturing and analyzing VOCs in environmental monitoring, indoor air quality assessments, and industrial emissions studies. Furthermore, whilst most polymer‐based adsorbents feature limited thermal stability (typically below 280 °C), the exceptional thermal stability of PPPO (≈450 °C) renders Tenax highly suitable for thermal desorption analysis applications.^[^ ^11^ ^]^ Both the EPA and NIOSH have specified the use of Tenax in their standard methods.

Tenax appears in a granular form, posing several significant limitations, including a relatively low specific surface area (≈25 m^2^ g^−1^) and restricted mass transfer within the packed granules, negatively impacting adsorption and desorption efficiency.^[^ ^26^, ^29^, ^30^ ^]^ In dynamic sampling, the use of Tenax‐packed columns is often accompanied by high‐pressure drops, limited flow rates and scalability issues. Moreover, factors such as granule agglomeration, heterogeneous pore sizes, and irregular structures further reduce the adsorption capacity of Tenax granules. These peculiarities also contribute to poor reproducibility during sample loading and analyte elution.^[^ ^31^ ^]^ A method for controlling the pore size and distribution of PPPO beads was recently proposed.^[^ ^32^ ^]^ It involves holding the droplets of PPPO solution aloft without mechanical support as they evaporate, resulting in beads with uniformly sized and distributed pore structures.

With membrane‐like adsorbents, there are several operational advantages, including the ability to operate at low‐pressure drops and high flow rates, as well as scalability. Forming membranes using electrospinning is a common method that enables flexible geometries, providing a simple and versatile approach to making highly efficient adsorbents.^[^ ^33^, ^34^, ^35^, ^36^, ^37^ ^]^ Membranes composed of nano/microfibers obtained by electrospinning are particularly interesting in usage as adsorption materials.^[^ ^38^, ^39^, ^40^, ^41^ ^]^ Electrospinning is a technique for producing fibers by applying a high electrical field to a polymer solution or melt, creating an electrically charged jet that stretches and solidifies as it travels to a grounded collector.^[^ ^42^ ^]^ This process enables the production of fibers with tunable porosity, high surface‐to‐volume ratios, and interconnected pore structures. These features enhance mass transfer, facilitate active site accessibility, and improve the functional performance of the material.^[^ ^43^, ^44^ ^]^ Precise control over fiber morphology, including its alignment, diameter and porosity, can be attained by adjusting process parameters, solution composition and environmental conditions.^[^ ^33^, ^34^, ^35^, ^36^, ^37^ ^]^

The composition of solvent mixtures exerts a critical influence on the electrospinning process and the structural properties of the resulting fibers, including their microstructure, diameter, uniformity, and porosity.^[^ ^45^, ^46^, ^47^, ^48^ ^]^ Polymer dissolution in solvent/non‐solvent systems induces phase separation via a non‐solvent‐induced phase separation (NIPS) mechanism, which is key to developing both core and surface porosity in electrospun fibers.^[^ ^49^, ^50^, ^51^, ^52^ ^]^ During electrospinning, the dramatic increase in the surface area of the jet results in solvent evaporation on a millisecond timescale.^[^ ^53^ ^]^ This rapid evaporation shifts the solvent mixture composition, facilitating phase separation into polymer‐rich and polymer‐lean phases, particularly when the solvent evaporates more quickly than the non‐solvent. Eventually, the polymer‐rich phase solidifies to form the fiber matrix, while the non‐solvent saturated phase changes into pores as the non‐solvent evaporates.^[^ ^52^, ^54^ ^]^ Environmental factors, such as humidity, further influence fiber porosity. High humidity enhances vapor‐induced phase separation (VIPS) by promoting water vapor condensation on the jet, resulting in interconnected pore networks or highly porous surfaces. These mechanisms could collectively shape the micro‐ and nanoscale features of electrospun fibers.^[^ ^54^, ^55^, ^56^, ^57^ ^]^

An electrospun PPPO fibrous membrane is presented in this work, which combines the exceptional properties of PPPO and the advantages of fibrous membranes. The resulting fibers exhibited porosity both in the core and the free surface, as indicated by their extensive surface area and textured surface structure. These properties and the intrinsic properties of fibrous membranes, such as permeability, make polymer fibers an ideal candidate for sampling volatile organic compounds (VOCs).

## 2 Results and Discussion

### 2.1 Morphological Characterization

**Figure 1:**
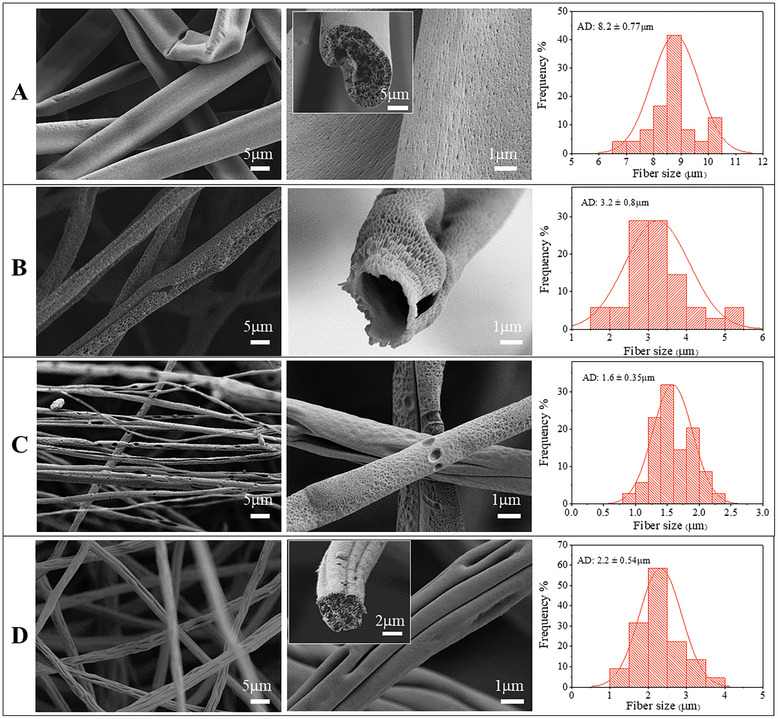
HRSEM images of fibers electrospun from different solvent mixtures (Table 1: **A**, **B**, **C**, **D**) and the corresponding fiber diameter distributions. AD denotes the average fiber diameter.

HRSEM images of PPPO fibers electrospun from various solvent and non‐solvent mixtures (**Table** **1**), along with their diameter distributions, revealed significant variations in morphology (**Figure** **1**). A macroscopic view of electrospun fibrous membranes is shown in Figure S1 (Supporting Information).

**Table 1:** List of the solvent mixtures for electrospun PPPO fibers fabrication. The solvents dielectric constants are ε_CF_ – 4.8; ε_THF_ – 7.58; ε_DMSO_ – 46.7; ε_EtOH_ – 24.6; ε_BuOH_ – 17.5. The description of the abbreviations is given in Experimental Section.

Solvent mixture	Composition of mixture	Component ratio,%v/v	Polymer concentration, %wt
A	CF/DMSO	85/15	7
B	CF/DMSO/EtOH	83/7/10	7
C	THF/DMSO	85/15	15
D	THF/DMSO/ BuOH	80/13/7	15

In most cases, fiber morphology was non‐uniform, except for the electrospun fiber membranes made from solvent mixture **A** (EM‐**A**), which demonstrated consistent and uniform fiber morphology with a significantly larger diameter than other fibers. Both surface and core porosity were strongly influenced by solvent composition. Fibers derived from EM‐**B** had a high level of surface porosity, while fibers derived from other mixtures had a high level of core porosity.

In surface porous fibers ambient humidity fluctuations had only a very small effect, EM‐**B** (Figure **S2**, Supporting information). Thus, NIPS appears to be the dominant mechanism driving core and surface porosity in PPPO electrospun fibers.

The PPPO fibers EM‐**A** (Figure 1: A) display a flat ribbon structure with fibers surface covered by a crust. Small pores/pits, ≈10–30 nm in diameter, were detected on the surface of the fibers. The cross‐section of these fibers revealed the core porosity. The fibers exhibited irregular shapes but uniformly distributed and interconnected mesopores. The formation of flat ribbon‐like fibers is generally caused by polymer solidification at the jet surface during electrospinning, which results in a skin layer (or shell). In some cases, solvents may be trapped within fiber shells. Significant pressure differences are created across the fiber shell when the core solvent evaporates from the solidified shell. As a result, fiber shells can buckle if the pressure drop across them exceeds a critical value; however, buckling is primarily determined by the rheological properties of the shell and core.^[^ ^58^, ^59^ ^]^ The resulting dense skin layer can impact the properties of the electrospun fibers, including their mechanical strength, porosity, and surface morphology. When working with solvent mixture **A**, the presence of the low‐volatile non‐solvent DMSO (boiling temperature (*T*_b_) 189 °C versus 61 °C for CF) in the fiber's core leads to skin formation and its collapse into flat fibers.

The addition of EtOH (solvent mixture **B**) as a supplementary non‐solvent significantly altered the morphology and size of the fibers (Figure 1: B). The resulting fibers, with an average diameter of 3.2 µm, exhibit a circular cross‐section, a porous surface with pore sizes ranging from 50–250 nm, and a hollow interior. The electrospinning stability, fiber diameter, and inter‐fiber distance were somewhat dependent on the CF/DMSO/EtOH ratio, the optimal value of which was determined experimentally. The prominent surface porosity observed in EM‐**B** was correlated to the comparable volatilities of the solvent CF and the non‐solvent component EtOH in the mixture (boiling points of 61 and 78 °C, respectively), suggesting that both solvents migrate toward the surface of the fibers, subsequently giving rise to surface porosity upon solvents evaporation. At the same time, low‐evaporating DMSO migrates more slowly to the jet surface and forms a polymer‐lean zone at the jet's core. These migration processes in the jet surface led to the formation of two‐phase regions in the fibers: the polymer‐rich (mostly in CF) and polymer‐lean (mostly consisting of EtOH) at the fiber surface^[^ ^41^, ^60^ ^]^ as well as polymer‐lean (mostly consisting of DMSO) in the core of the fiber. The further evaporation of the mixture components gives rise to porosity on the fiber surface and can lead to the formation of a hollow interior.^[^ ^51^, ^54^, ^60^, ^61^ ^]^

In Figure 1C, the surface porosity and interior porosity of fibers prepared from solvent mixture **C** are shown. Pronounced mesopores (diameter 2–50 nm), and a few macropores (>50 nm) are visible, opening to the outside surface of the fibers. Compared to fiber electrospun from solvent mixture **B**, these fibers have significantly smaller pores. The formation of interior porosity can be attributed to the considerably slower evaporation of DMSO in comparison to THF, leading to an increased portion of non‐solvent DMSO within the core of the fibers. As the solvent evaporates, the polymer‐lean region develops, the inner fiber porosity increases, and the free surface pores are connected. The mechanism of this phase separation involves the nucleation and growth of a specific phase, confirmed by the presence of isolated pores on the surface of the fibers.^[^ ^62^ ^]^

Adding BuOH, which has a much higher boiling point ‐ 117 C, than EtOH, to solvent mixture **C**, resulting in mixture **D**, significantly stabilizes electrospinning and gives the fibers a specific surface morphology. A relatively low surface porosity can be found on the grooved, longitudinal fibers (i.e., radial buckling) (Figure 1D). The internal porosity of these fibers can be seen in detail in the figure inset. The observed superficial grooves are oriented along the fiber axis, which may be attributed to the mechanism of phase separation^[^ ^42^ ^]^ when the electrospun solution jet is still incompletely solidified^[^ ^63^ ^]^ and the residual DMSO, concentrating in the jet core, facilitating the wrinkled jet's surface to be stretched into a grooved texture.^[^ ^64^ ^]^

### 2.2 Specific Surface Area Characterization (BET)

Brunauer‐Emmett‐Teller (BET) analysis was used to estimate the specific surface area of the samples.^[^ ^65^ ^]^ Compared to the standard granular Tenax, electrospun fibrous membrane EM‐**A** showed a nearly three‐fold increase in specific surface area. Interestingly, fibers with smooth surfaces and internal porosity had the highest specific surface area, while fibers with pitted surfaces did not (**Table** **2**).

**Table 2:** The specific surface area (SSA) of electrospun fibers obtained from different electrospinning solutions (A, B, C, D) and granular Tenax. Standard deviations were calculated based on the results of triplicate testing.

Electrospun membrane (EM) – solvent mixture (see Table 1)	BET surface area [m^2^ g^−1^]
EM‐A	70 ± 1.4
EM‐B	52 ± 1.7
EM‐C	19 ± 0.57
EM‐D	28 ± 0.65
Granular Tenax	24.6 ± 0.7

### 2.3 Thermal and Structural Characterization of PPPO Electrospun Fibers

**Figure 2:**
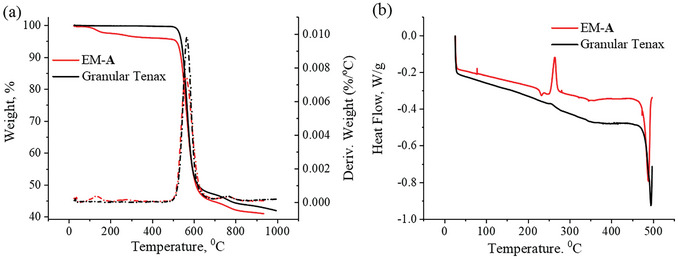
a) Thermogravimetric (TGA) and derivative thermogravimetric (DTG) curves of EM‐**A** fibers and granular Tenax. b) DSC thermographs of EM‐**A** and granular Tenax.

Thermal stability is a critical property for adsorbent materials, particularly for thermal desorption applications, which require operation at elevated temperatures (≈350 °C). TGA and DSC analyses were performed to assess the impact of the electrospinning process on the thermal properties of the obtained fibers, including their degradation and melting temperatures. As a representative sample material for thermal and structural characterizations, fibrous membrane EM‐A was selected. The derivative thermogravimetric (DTG) analysis of the electrospun fibers exhibited the onset of the thermal decomposition peak at 505 °C, comparable to that observed for Tenax granules (**Figure** **2**a). The degradation peak of electrospun fibers and granular Tenax appeared at 561 and 568 °C, respectively. The DSC curves of electrospun fibers and granular Tenax are shown in Figure 2b. The PPPO fibers exhibited two distinct thermal events: an endothermic peak at 489 °C, corresponding to the melting temperature, and an exothermic peak at 264 °C, associated with cold crystallization. This crystallization phenomenon has been previously reported in electrospun fibers and is attributed to a glass‐like polymer chain state induced by fast solvent evaporation and cooling during the electrospinning process, which further facilitates polymer crystallization upon heating.^[^ ^66^ ^]^ Contrarily, granular Tenax exhibited only the melting peak, at 495 °C, designating a stable semi‐crystalline structure of the granules. Furthermore, the electrospun fibers, EM‐**A**, displayed a slightly lower glass transition temperature (*T*_g_ ≈228 °C), compared to granular Tenax (*T*_g_ ≈250 °C). The lower *T*_g_ of electrospun fibers compared to polymer films or beads has been previously reported.^[^ ^67^, ^68^ ^]^ This phenomenon was attributed to several factors, such as higher surface area, inner stress, and a high degree of orientation of polymer chains during electrospinning.

Ultimately, the TGA and DSC analyses demonstrated that the electrospinning process had a minor effect on the thermal properties of the polymer, ensuring that the polymer retains its physical properties under heat.

**Figure 3:**
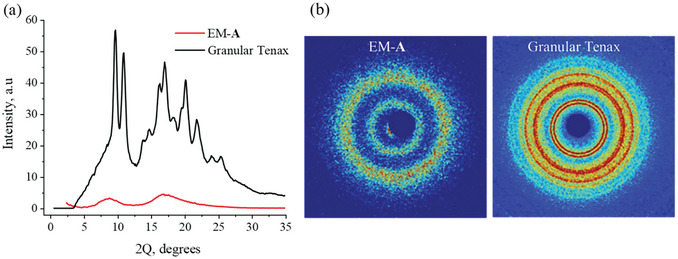
a) WAXS diffraction pattern of the electrospun PPPO fibers (EM‐**A**), and granular Tenax (averaging over azimuth of 360 degrees). b) 2D X‐ray diffraction patterns of the samples.

WAXS measurements were performed to evaluate the microstructure of PPPO electrospun fibers (**Figure** **3**). The WAXS diffraction pattern of EM‐**A** fibers displayed an amorphous halo pattern, suggesting the as‐spun fibers were fully amorphous (Figure 3b) and had no molecular orientation in the fibers. In contrast, the thermographs of commercial granules revealed pronounced crystalline peaks. These findings confirmed the results obtained by DSC. As was previously shown, PPPO films characterized by lower crystallinity displayed higher adsorption capacity.^[^ ^69^, ^70^ ^]^ Additionally, it has been demonstrated that the adsorption of analytes in PPPO occurred essentially in the amorphous phase. SAXS results of the samples are depicted in Figure S3 (Supporting Information) and indicate a homogeneous pore population in a nanometer range.

### 2.4 VOCs Sampling

There are two general ways to implement VOC sampling: passively or dynamically. Dynamic sampling involves actively drawing air through a sampling device to collect VOCs., while passive air samplers rely on the diffusion of VOC into an adsorbent over time. Choosing the right sampling method depends on the VOCs of interest, the expected concentration range, the required sensitivity, accuracy, and the type of sampling method (grabs, time‐integrated, or real‐time).

#### 2.4.1 Passive Sampling

**Figure 4:**
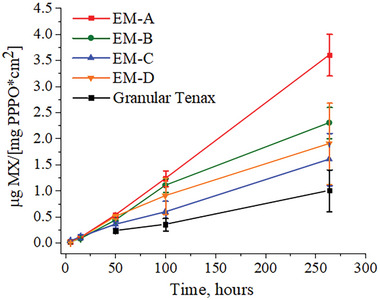
Time‐resolved passive adsorption of MX onto various electrospun PPPO‐based fibrous membranes and granular Tenax. Standard deviations were calculated based on the results of triplicate testing of two independently prepared membranes.

The kinetics of MX adsorption on electrospun PPPO membranes and commercial granular Tenax are depicted in **Figure** **4**. All tested PPPO electrospun membranes demonstrated higher adsorption capacity compared to granular Tenax, attributed to larger surface area and greater accessibility of adsorption sites in membranous samplers. Among the PPPO membranes, ES‐**A** exhibited superior adsorption capacity, ascribed to higher specific surface area and predominantly mesoporous core porosity, which is expected to enhance the adsorption capacity. The lower adsorption capacity of EM‐**B** is likely due to its uppermost surface porosity and hollow fiber interior. Sample EM‐**D** exhibited higher adsorption capacity than EM‐**C**, likely due to higher specific surface area. It should be noted that within the experimental timeframe, the adsorption process did not reach equilibrium.

#### 2.4.2 Dynamic Sampling

An electrospun PPPO membrane, type EM‐**A**, was compared to a granular Tenax column to assess its dynamic sampling efficiency. The membranous sampler has a diameter of 2.5 cm. The average pore was 45 ± 25 µm with an overall porosity of 88 ± 4% and permeability of ≈10^−13^ m^2^. Note that the permeability of the Tenax column was of ≈10^−11^ m^2^.^[^ ^71^ ^]^

It is well‐established that adsorption efficiency decreases as the superficial velocity increases due to the reduced retention time of molecules within the sampler.^[^ ^72^ ^]^ To ensure similar superficial velocity the flow rates were selected as: 10 and 1 L min^−1^ for the EM‐**A** and Tenax‐bead column, respectively. The mass of MX adsorbed onto the electrospun membrane (EM‐**A**) and Tenax column was 3.9 ± 0.5 and 0.54 ± 0.16 (ng MX/mg adsorbent), respectively.

The PPPO membrane showed approximately sevenfold higher adsorption capacity than the granular Tenax.

**Figure 5:**
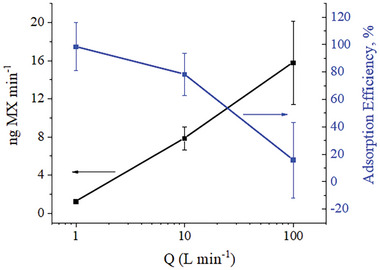
The amount of MX adsorbed onto EM‐**A** and adsorption efficiency as a function of different operating flow rates, Q. A standard deviation based on three measurements is shown in the error bars.

Additionally, the application of PPPO membranes for air sampling at flow rates up to 100 L min^−1^ was demonstrated. **Figure** **5** illustrates the mass of adsorbed MX and the corresponding adsorption efficiency across different flow rates, ranging from 1 to 100 L min^−1^. The amount of adsorbed MX increased with flow rates from 1 to 100 L min^−1^; however, a notable decline in adsorption efficiency was detected. While adsorption efficiency was ≈80% at 10 L min^−1^, it decreased to 15% at 100 L min^−1^, indicating a significant loss of target molecules during sampling. This decrease was attributed to the significantly higher superficial velocity within the sampler (≈ 5 m s^−1^), which impeded the molecules from reaching the adsorption sites.

## 3 Conclusion

This study demonstrated the electrospinning of PPPO polymer from different solvent/nonsolvent mixtures for the preparation of VOC sampling membranes. Highly porous PPPO fibers were produced by electrospinning a solution of polymer in binary (CF or THF)/DMSO or ternary (CF or THF)/DMSO/(EtOH or BuOH) solvent mixtures in a straightforward way. Experimental results demonstrate that porous fibers can be easily achieved if the solvent is more volatile than the non‐solvent in the spinning solution. Moreover, the fibers' morphology, specifically their diameter and porosity, can be controlled by manipulating the solvent/non‐solvent type and ratio. The presence of DMSO in all formulations was critical to prevent massive evaporation of the solution adjacent to the spinneret and to increase the dielectric constant of the solution, thereby ensuring a stable jet.

PPPO fibers' enhanced surface area and porous morphology resulted insuperior adsorption, both passively and dynamically compared to granular Tenax  . The feasibility of PPPO membrane for sampling at flow rates up to 100 L min⁻¹ was demonstrated, although a notable decrease in adsorption efficiency was observed. To improve the performance of these membranes for high‐flow‐rate VOC sampling (>100 L min⁻¹), various geometric configurations of the samplers should be explored. Also, assessing the applicability and efficiency of PPPO electrospun membranes in thermal desorption analysis methods remains a crucial focus for further study.

## 4 Experimental Section

### Materials

TenaxTA granules, 80–100 mesh, and TenaxTA cartridge (ORBO 402) were purchased from Sigma‐Aldrich, Ltd. Chloroform (CF), tetrahydrofuran (THF), dimethyl sulfoxide (DMSO), butanol (BuOH), acetone (≥99.8%) and ethanol (EtOH) were supplied by Merck. Musk Xylene (MX) was obtained from Puyang Yuantai Fine Chemicals Co., Ltd.

### Characterization Methods

High‐resolution SEM (HRSEM) images of the electrospun fibers were obtained using Carl Zeiss Ultra Plus HRSEM. The samples were carbon‐coated before imaging. The accelerating voltage of 1 keV was applied. A secondary electron detector was used to study morphology and surface topography.

Thermal gravimetric analysis (TGA) was realized on Mettler Toledo TGA‐Q5000 (TA instruments, USA). The measurements were used to characterize the thermal stability of the specimens. The tests were conducted under a nitrogen atmosphere at a heating rate of 10 °C min^−1^ in the temperature range of 25–800 °C.

Differentiated scanning calorimetry (DSC) (Discovery DSC (Q2000), TA Instruments Inc.) was used to study the thermal behavior of the samples. The scan intervals were 25–500 °C, with the heating rate of 5 °C min^−1^. Duplicate analysis was conducted for the hermetically sealed in the aluminum pan samples.

Gas Chromatography‐Mass Spectrometry (GC‐MS) samples were analyzed and quantified using a 6890N GC instrument (Agilent Technologies, CA, USA) equipped with a capillary HP5‐MS column (60 m × 250 µm × 0.25 µm, Agilent Technologies) and 5975 Mass Selective Detector (MSD) system (Agilent Technologies, CA, USA). Helium was used as a carrier gas.

Small‐angle and wide‐angle x‐ray spectroscopy (SAXS and WAXS, respectively) measurements were performed using a small/wide‐angle diffractometer (Molecular Metrology) with CuKα radiation from a sealed micro‐focus tube (MicroMax‐002+S), two Göbel mirrors, three‐pinhole slits, and generator powered at 45 kV and 0.8 mA.

The specific surface area (SSA) of the samples was estimated using FlowSorb II 2300 (Micromeritics, GA, USA) analyzer. The fibrous mats were degassed at temperatures between 140–180 °C, and then N_2_ physisorption at 77K was used to measure their SSA.

### Experimental Methods

PPPO was dissolved in various solvent and non‐solvent mixtures, with polymer concentrations fixed at 7 and 15 wt.%. Continuous stirring at room temperature produced homogeneous and transparent polymer solutions. As volatile solvents, CF and THF were chosen due to their excellent solubility properties. To enhance the electrical conductivity of the prepared solutions and to control the fiber's morphology, high dielectric constant co‐solvents/non‐solvents: DMSO, EtOH, and BuOH, were incorporated. The addition of these solvents, particularly DMSO, due to its relatively low volatility, hinders plug formations in the needle and improves process stability.

The PPPO solution was delivered to the tip of the needle by a syringe pump Harvard Apparatus, Pump 11 Elite, with a feeding rate of ≈1.5 mL h^−1^ at room temperature. A positive high voltage of 7–10 kV was then applied to the polymer solution via the stainless‐steel syringe needle. Electrospun fibers were deposited on the surface of a rotating drum (Al 6061) positioned 7 cm from the needle tip, forming a porous fiber nonwoven mat.

Adsorption efficiency was studied using two sampling approaches: passive and dynamic. Passive air sampling relies on the diffusive uptake of chemical vapor into a sorbent over time, while dynamic sampling involves drawing air through or across an adsorbent using a pump, allowing precise control over flow rate and sampling time.^[^ ^73^, ^74^ ^]^ To evaluate the amount of adsorbed analyte on various samplers, a solvent extraction method was chosen.

### Passive Sampling

To assess the adsorption capability of various PPPO electrospun membranes and granular Tenax, Musk Xylene (MX), a semi‐volatile compound (P_sat_ ≈10^−10^ atm), was selected as a representative analyte. MX powder, ≈5 g, was deposited at the bottom of a 0.5 L glass vessel. The electrospun membranes and commercial granules were mounted on a support grid and positioned above the analyte source. A time‐resolved experiment was performed with samples collected over a period of 5 to 264 h to evaluate the adsorption kinetics. At each time interval, the samples were removed and soaked in acetone for two hours. The quantification of the adsorbed MX was performed using GC‐MS.

### Dynamic Sampling

The dynamic sampling efficiency of electrospun membranes was compared with that of Tenax cartridges. A metal‐based container (32 m^3^) was filled with 300 g of MX. Using Tenax cartridges operating at 0.25 L min^−1^, after 10 days, the steady‐state concentration of MX was 1.22 ng L^−1^. Sampling was carried out using membranes at flow rates of 1, 10, and 100 L min^−1^ and with the Tenax cartridges at 1 L min^−1^. The total amount of MX adsorbed on both types of samplers was then determined using solvent extraction method followed by GC‐MS analysis. Adsorption efficiency (%) was determined as the ratio of the mass of the substance adsorbed onto the adsorbent and the initial mass of the MX in the container (1.22 ng L^−1^).

## Conflict of Interest

The authors declare no conflict of interest.

## Supporting information



Supporting Information

## Data Availability

The data that support the findings of this study are available in the supplementary material of this article.
