# Supplementary material for: Tenax‐Based Electrospun Fibrous Membranes for Efficient VOC Sampling
Source: Macromol Rapid Commun. 2025 Mar 3;46(13):2400969. doi: 10.1002/marc.202400969 (PMC12227226; doi:10.1002/marc.202400969)
Supplement: Supplementary file 1 — Supporting Information [file MARC-46-2400969-s001.docx]

**Supporting Information**

**TENAX-based electrospun fibrous membranes for efficient VOC sampling**

R.Vilensky, O. Marom, D. M. Rein, E. Zussman*

**Figure S1:** Electrospun fiber mats produced from PPPO with the following solvent mixture: A- CF/DMSO, B- CF/DMSO/EtOH, C- THF/DMSO, and D- THF/DMSO/ BuOH.


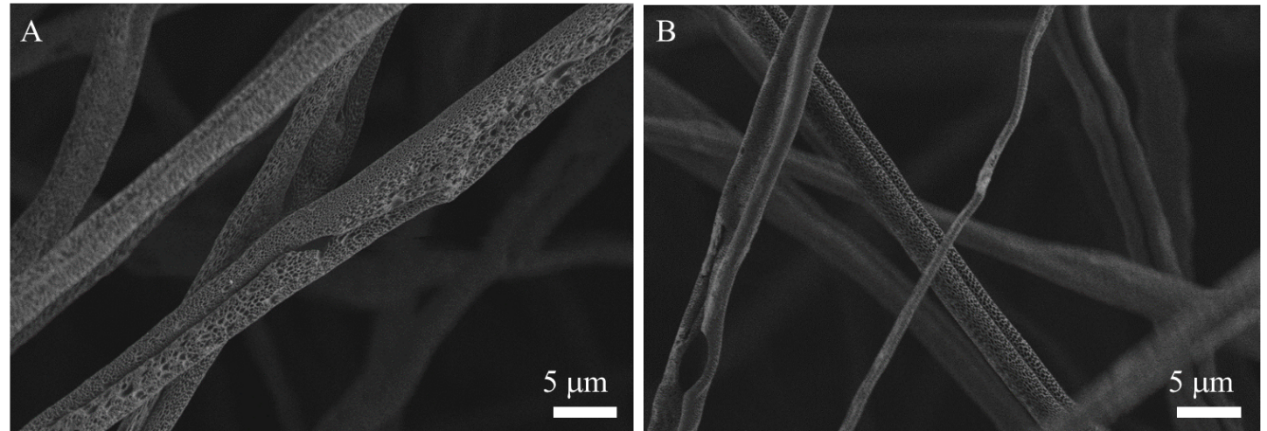


**Figure S2:** Fibers produced from chloroform/DMSO/EtOH solution electrospun under varying humidity conditions: A) 20%, and B) 80-90 % (obtained by using a humidifier in the hood).

The SAXS results of the samples are depicted in **Figure S3**. It can be seen that in the double-logarithmic plot, the curves follow Porod's law.^[1]^ Namely, the intensity *I*(*h*) is proportional to *h*^−4^ for larger values of *h*. The results indicate a homogeneous pore population in a nanometer range.

**Figure S3**: Double-logarithmic plot of the scattered intensity I(h) at the interval 0.07 < h < 2.7nm^−1^, where h is the scattering vector and the radiation wavelength (0.1542 nm) of as-spun fiber samples EM-**A**, annealed EM-**A** and granular Tenax-TA.

_____________________________

[1] G. Porod, in *General Theory*, (Eds.: O. Glatter, O. Kratky), Academic

Press, Cambridge, **1982**, *Small Angle X-ray Scattering*, pp. 17–51.
